# Enhanced immune response of MAIT cells in tuberculous pleural effusions depends on cytokine signaling

**DOI:** 10.1038/srep32320

**Published:** 2016-09-02

**Authors:** Jing Jiang, Xinchun Chen, Hongjuan An, Bingfen Yang, Fuping Zhang, Xiaoxing Cheng

**Affiliations:** 1 Shenzhen Key Laboratory of Infection and Immunity, Shenzhen Third People’s Hospital, Guangdong Medical College, Shenzhen, Guangdong, China; 2 Division of Research, Institute of Tuberculosis, 309th Hospital, Beijing, China; 3 Institute of Microbiology, Chinese Academy of Sciences, Beijing, China; 4 Department of Pathogen Biology, School of Medicine, Shenzhen University, Shenzhen, Guangdong, China

## Abstract

The functions of MAIT cells at the site of *Mycobacterium tuberculosis* infection in humans are still largely unknown. In this study, the phenotypes and immune response of MAIT cells from tuberculous pleural effusions and peripheral blood were investigated. MAIT cells in tuberculous pleural effusions had greatly enhanced IFN-γ, IL-17F and granzyme B response compared with those in peripheral blood. The level of IFN-γ response in MAIT cells from tuberculous pleural effusions was inversely correlated with the extent of tuberculosis infection (p = 0.0006). To determine whether cytokines drive the immune responses of MAIT cells at the site of tuberculosis infection, the role of IL-1β, IL-2, IL-7, IL-12, IL-15 and IL-18 was investigated. Blockade of IL-2, IL-12 or IL-18 led to significantly reduced production of IFN-γ and/or granzyme B in MAIT cells from tuberculous pleural effusions. Majority of IL-2-producing cells (94.50%) in tuberculous pleural effusions had phenotype of CD3^+^CD4^+^, and most IL-12p40-producing cells (91.39%) were CD14^+^ cells. MAIT cells had significantly elevated expression of γc receptor which correlated with enhanced immune responses of MAIT cells. It is concluded that MAIT cells from tuberculous pleural effusions exhibited highly elevated immune response to *Mtb* antigens, which are controlled by cytokines produced by innate/adaptive immune cells.

Tuberculosis (TB) is the second leading cause of death from an infectious disease worldwide. It is estimated that 9.0 million people developed TB in 2013 and 1.5 million died from the disease in the world^1^. *Mycobacterium tuberculosis (Mtb*), the causative agent of TB, most often infects the lungs in humans, but it can infect other organs and cause extrapulmonary TB. Tuberculous pleurisy is one of the most common forms of extrapulmonary TB on global perspectives, and might be responsible for 30–80% of all pleural effusions in some developing countries^2^,^3^,^4^. It is now believed that tuberculous pleurisy is the consequence of direct *Mtb* infection of the pleural space in humans^2^.

Mucosal-associated invariant T (MAIT) cells are innate-like T cells that play an important role in protective immunity against microbial infections, most likely through production of effector molecules, including INF-γ, TNF-α, IL-17, and granzyme B^5^,^6^,^7^,^8^,^9^,^10^. MAIT cells display a semi-invariant T cell receptor (TCR) α chain that consists of TRAV1-2 gene paired with different TRAJ genes, including TRAJ33, TRAJ12 and TRAJ20^11^,^12^,^13^,^14^,^15^, and recognize microbial vitamin B metabolites presented by the major histocompatibility complex (MHC)-like molecule MR1^11^,^16^,^17^,^18^. MR1-antigen tetramers identify mouse MAIT cells in broad range of tissues with heterogeneous phenotypes, including CD4^−^CD8^−^, CD4^−^CD8^+^ and CD4^+^CD8^−^ subsets^19^,^20^. MAIT cells are abundant in humans, including peripheral blood, liver, gut lamina propria and lungs^5^,^6^,^21^,^22^.

It is proven that MAIT cells can protect against infection by *Mycobacterium abscessus* in mice^5^, and potently inhibit growth of *Mycobacterium bovis* BCG in macrophages^23^. In mycobacterial pulmonary infection of mice, MAIT cells are recruited into the lungs and provide early protection^20^. In humans, the frequency of MAIT cells are decreased in peripheral blood from patients with active TB^5^,^6^,^24^,^25^, but are enriched in human lung and in ascitic fluids from patients with tuberculous peritonitis^6^,^24^.

In humans, MAIT cells in peripheral blood have relatively poor cytokine response to *Mtb* antigens in comparison to other bacterial infection^24^,^25^. It is not clear whether MAIT cells in humans at the site of TB infection have different phenotypes and immune response to *Mtb* antigens. In this study, we investigated the phenotypes and immune response of MAIT cells in pleural effusions from patients with tuberculous pleurisy, and found that MAIT cells in tuberculous pleural effusions, the site of TB infection, had greatly enhanced IFN-γ, IL-17F and granzyme B response compared with those in peripheral blood. The enhanced production of cytokine and cytotoxic effector in MAIT cells from tuberculous pleural effusions was dependent on IL-2 produced predominantly by CD4^+^ T cells and/or IL-12 produced mainly by CD14^+^ cells.

## Results

### MAIT cells from tuberculous pleural effusions exhibited elevated IFN-γ response to *Mtb* antigens

**Figure 1:**
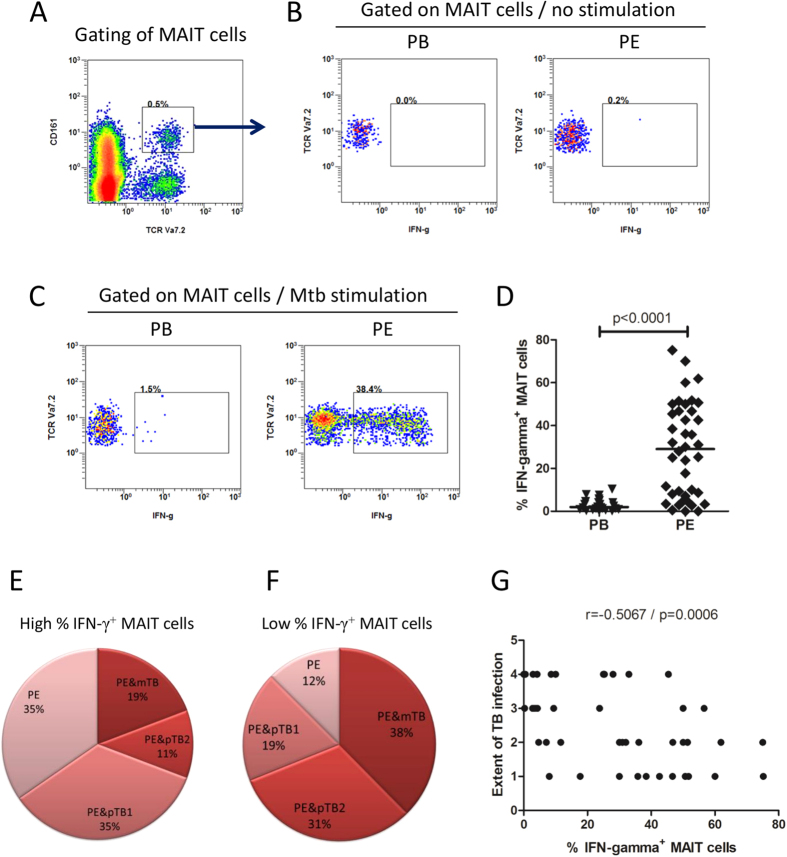
IFN-γ production in MAIT cells from tuberculous pleural effusions and peripheral blood. (**A**) Representative flow cytometric plot showing gating of MAIT cells with phenotypes of CD3^+^Vα7.2^+^CD161^high^. (**B**) Representative flow cytometric plots showing IFN-γ production in MAIT cells from peripheral blood (PB) and from tuberculous pleural effusion (PE) of patient with tuberculous pleurisy in the absence of antigen stimulation. (**C**) Representative flow cytometric plots showing IFN-γ production in MAIT cells from peripheral blood (PB) and from tuberculous pleural effusion (PE) of patient with tuberculous pleurisy after *Mtb* antigen stimulation. (**D**) MAIT cells in tuberculous pleural effusions had greatly enhanced IFN-γ response to *Mtb* antigens compared with those in peripheral blood. Horizontal Bars in the scatter plots indicate median. (**E**) The ratio of patients with tuberculous pleurisy only (PE), with accompanied pulmonary TB with lesions located within one lung (PE & pTB1), with accompanied pulmonary TB with lesions located in both lungs (PE & pTB2), and with accompanied pulmonary TB and other extrapulmonary TB (PE & mTB) in the high IFN-γ-producing MAIT cell group. (**F**) The ratio of patients with different extent of TB infections in the low IFN-γ-producing MAIT cell group. (**G**) Correlation analysis showed that the frequency of IFN-γ-producing MAIT cells was inversely correlated with the extent of TB infection. The extend of TB infection was graded from 1 to 4, representing PE, PE&pTB1, PE&pTB2 and PE&mTB respectively, as described in legend in Fig. 1E. The nonparametric Mann-Whitney test was used for statistical analysis in Fig. 1D.

Previous investigations on human MAIT cells in patients with active TB are mainly focused on cells from peripheral blood. It is postulated that MAIT cells from *Mtb* infection sites might have different phenotypes and functional properties. In this study, we recruited 42 patients with tuberculous pleurisy (Table 1), and compared phenotypes and functional characteristics of MAIT cells from tuberculous pleural effusions and peripheral blood. The mean percentages of CD14^+^ monocytes/macrophages, CD19^+^ B cells, CD4^+^ and CD8^+^ T cells in purified cells from tuberculous pleural effusions were 6.89%, 8.95%, 59.64% and 23.65% respectively, and the frequency of MAIT cells in the T cell population was 0.91%, as measure by flow cytometry. MAIT cells in tuberculous pleural effusions from patients with tuberculous pleurisy had greatly elevated IFN-γ response to *Mtb* antigens compared with those in peripheral blood (p < 0.0001) (Fig. 1A–D).

As shown in Fig. 1D, patients with tuberculous pleurisy can be divided into two groups, based on cut-off value of 20% IFN-γ production levels in MAIT cells. The high IFN-γ-producing MAIT cell group contained 26 patients and the low IFN-γ-producing MAIT cell group had 16 patients. There was no significant difference in age, and in adenosine deaminase (ADA) and lactate dehydrogenase (LDH) level, total proteins, glucose concentration and ratio of mononuclear cells in pleural effusions between the two groups. The frequency of MAIT cells in tuberculous pleural effusions did not show significant difference among patients of tuberculous pleurisy with different extent of TB infection (p = 0.6831).

To know whether level of IFN-γ response is related to disease progress of TB, the clinical characteristics of patients with tuberculous pleurisy were compared between the two groups. In the high IFN-γ-producing MAIT cell group, around 70% of patients had only tuberculous pleurisy or had accompanied lesions located within one lung, and only 30% of patients had TB lesions that affected both lungs or had other extrapulmonary TB (Fig. 1E). In contrast, in the low IFN-γ-producing MAIT cell group, only 31% patients had only tuberculous pleurisy or with lesions located within one lung, and 69% had greater range of TB infection (Fig. 1F).

We performed correlation analysis and found that frequency of IFN-γ-producing MAIT cells was not correlated with age and sex of the patients, levels of ADA, LDH, total proteins, glucose concentration and ratio of mononuclear cells in pleural effusions. However, it was inversely correlated with the extent of TB infection (r = −0.5067, p = 0.0006) (Fig. 1G).

To determine the role of MR1 in MAIT cell IFN-γ production, MR1 blocking antibody was added during *Mtb* stimulation. Addition of anti-MR1 antibody does not have significant effect on IFN-γ production in MAIT cell from tuberculous pleural effusions when stimulated with *Mtb* lysates (data not shown).

Taken together, the result suggested that the level of IFN-γ response in MAIT cells from tuberculous pleural effusions might inversely correlate with disease severity of TB.

### Elevated cytotoxic and IL-17 response in MAIT cells from tuberculous pleural effusions

**Figure 2:**
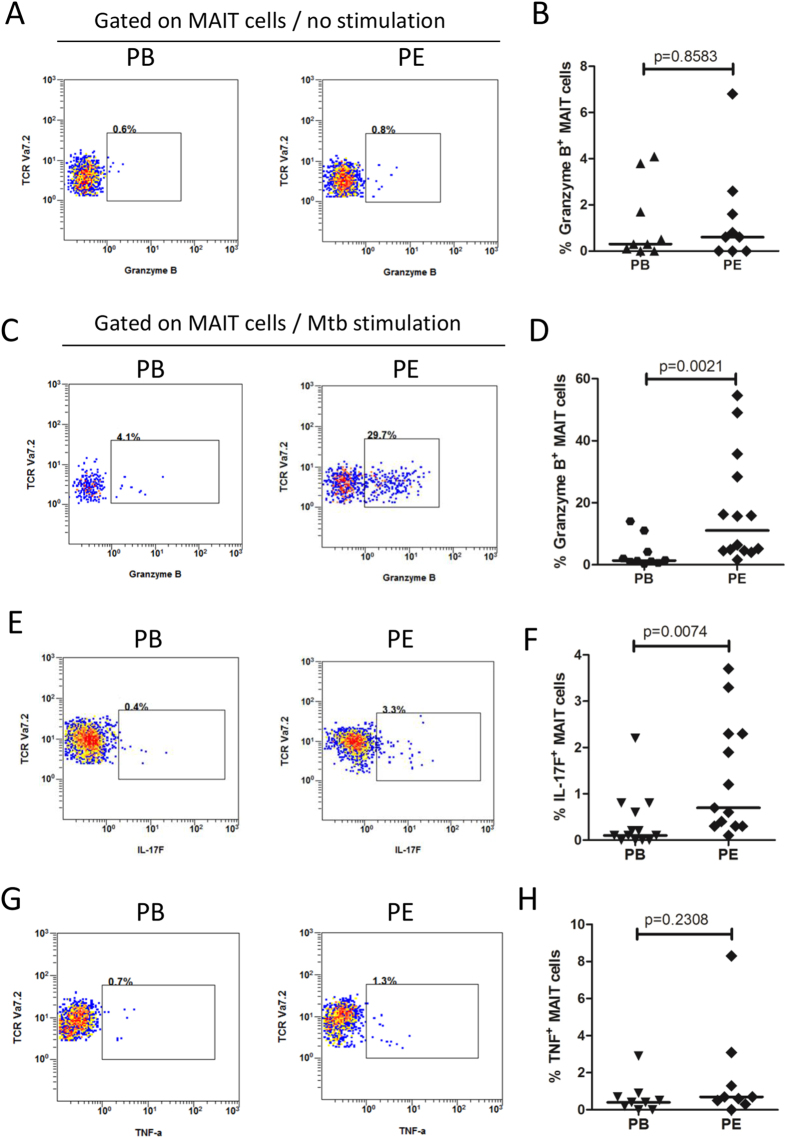
Expression of granzyme B, IL-17F and TNF-α in MAIT cells. (**A**) Representative flow plots showing expression of granzyme B in MAIT cells from peripheral blood (PB) and tuberculous pleural effusion (PE) in the absence of antigen stimulation. (**B**) MAIT cells from tuberculous pleural effusions had similar expression of granzyme B compared with those from peripheral blood in the absence of antigen stimulation. (**C**) Representative flow plots showing expression of granzyme B in MAIT cells from peripheral blood (PB) and tuberculous pleural effusion (PE) after *Mtb* antigen stimulation. (**D**) MAIT cells from tuberculous pleural effusions had much higher expression of granzyme B than those from peripheral blood after stimulation with *Mtb* lysates. (**E**) Representative flow plots showing expression of IL-17F in MAIT cells from peripheral blood (PB) and tuberculous pleural effusion (PE) after *Mtb* antigen stimulation. (**F**) MAIT cells from tuberculous pleural effusions had significantly higher expression of IL-17F than from peripheral blood. (**G**) Representative flow plots showing expression of TNF-α in MAIT cells. (**H**) TNF-α expression in MAIT cells did not show significant difference between the two groups. Horizontal bars in the scatter plots indicate medians. The nonparametric Mann-Whitney test was used for statistical analysis between groups in Fig. 2B,D,F,H.

The anti-microbial function of MAIT cells depends on production of effector molecules. To know whether other effector molecules besides IFN-γ are involved, we investigated expression of granzyme B, IL-17F and TNF-α in MAIT cells from tuberculous pleural effusions and peripheral blood of patients with TB by flow cytometry. Low levels of granzyme B expression were found in MAIT cells from both tuberculous pleural effusions and peripheral blood in the absence of antigen stimulation, and their frequency was similar (p = 0.8583) (Fig. 2A,B). After stimulation with *Mtb* lysates, MAIT cells from tuberculous pleural effusions had much higher expression of granzyme B than those from peripheral blood (p = 0.0021) (Fig. 2C,D). The production of IL-17F was also higher in MAIT cells from tuberculous pleural effusions (p = 0.0074) (Fig. 2E,F). However, TNF-α expression in MAIT cells was low and did not have significant difference between the two groups (p = 0.2308) (Fig. 2G,H). We also checked IL-17A and IL-22 production in MAIT cells by intracellular staining with specific antibodies and flow cytometry, and both of them were minimal in MAIT cells but were clearly detected in other cells from pleural effusions (data not shown).

Next we investigated the relationship between IFN-γ and granzyme B in MAIT cells from tuberculous pleural effusions by flow cytometry. IFN-γ production was significantly correlated with granzyme B expression in MAIT cells (p < 0.0001), and most IFN-γ-producing MAIT cells also expressed granzyme B (data not shown).

### Phenotypic characteristics of MAIT cells from tuberculous pleural effusions and peripheral blood

**Figure 3:**
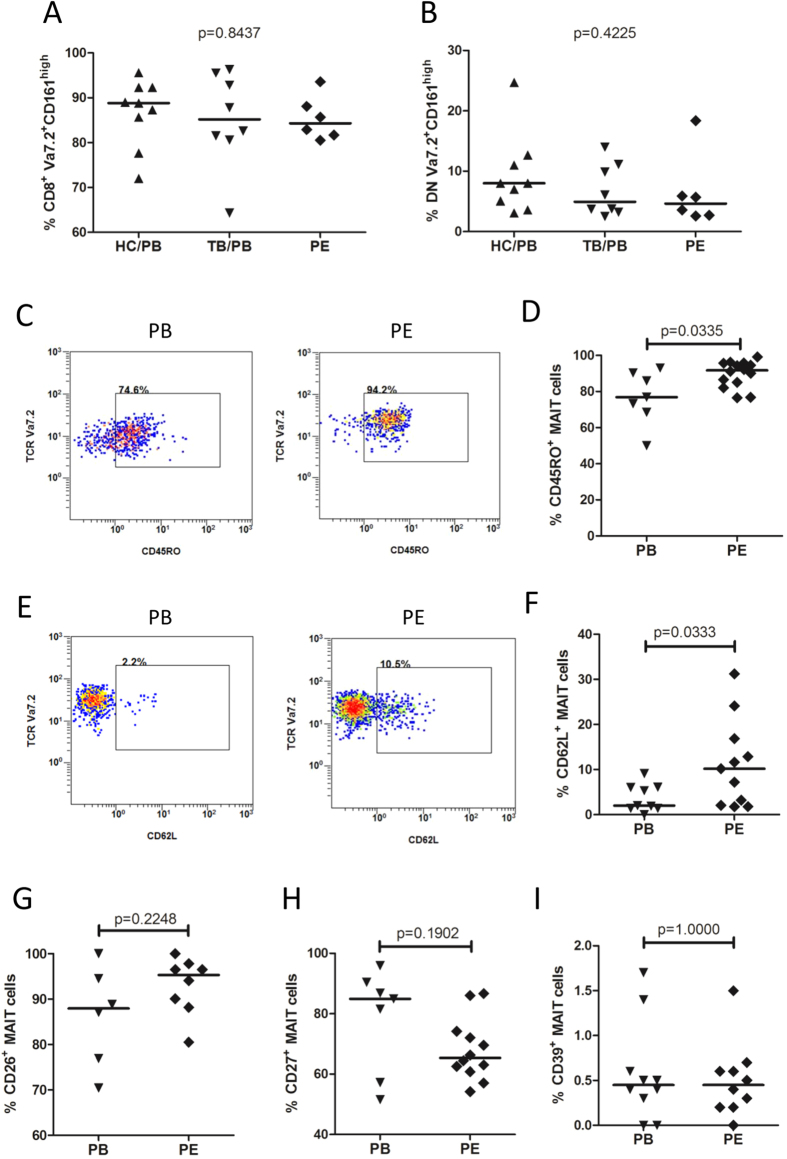
Phenotypic analysis of MAIT cells from tuberculous pleural effusions and peripheral blood. (**A**) Frequency of CD8α^+^ MAIT cells in peripheral blood of healthy controls (HC/PB) and patients with TB (TB/PB), and in tuberculous pleural effusions (PE). (**B**) Frequency of CD4^−^CD8α^−^ (DN) MAIT cells. (**C**) Representative flow plots showing expression of CD45RO in MAIT cells from peripheral blood (PB) and tuberculous pleural effusion (PE) in the absence of antigen stimulation. (**D**) Frequencies of CD45RO^+^ MAIT cells. (**E**) Representative flow plots showing expression of CD62L in MAIT cells in the absence of antigen stimulation. (**F**) Frequencies of CD62L^+^ MAIT cells. (**G**–**I**) Frequencies of CD26^+^ (**G**), CD27^+^ (**H**), CD39^+^ (**I**) MAIT cells in peripheral blood of patients with TB (PB) and in tuberculous pleural effusions (PE) respectively. Horizontal bars in the scatter plots indicate medians. Kruskal-Wallis test was used for statistical analysis among groups in Fig. 3A,B. The nonparametric Mann-Whitney test was used for statistical analysis between groups in Fig. 3D,F,G–I.

Next we compare phenotypes of MAIT cells from tuberculous pleural effusions and peripheral blood. CD3^+^Vα7.2^+^CD161^high^ cells from tuberculous pleural effusions and peripheral blood of patients with TB and healthy controls were further subtyped based on CD4 and CD8α expression. In general, majority of CD3^+^Vα7.2^+^CD161^high^ cells were CD8α^+^, a small number of cells were CD4^−^CD8α^−^ double negative, and very few cells were CD4^+^ (Fig. 3A,B). The percentage of CD8α^+^ (p = 0.8437) and CD4^−^CD8α^−^ cells (p = 0.4225) in tuberculous pleural effusions and peripheral blood from patients with TB was very similar (Fig. 3A,B). The result demonstrated that the vast majority of CD3^+^Vα7.2^+^CD161^high^ cells were CD8α^+^ and CD4^−^CD8α^−^ cells, and there was no difference of MAIT cell CD8/CD4 subtypes in peripheral blood and pleural effusions during TB infection.

CD45RO and CD62L were used to identify memory T cells in combination with other markers. MAIT cells in tuberculous pleural effusions had significantly higher expression of CD45RO (p = 0.0335) (Fig. 3C,D) and CD62L (p = 0.0333) (Fig. 3E,F) than in peripheral blood. The expression of CD26, CD27, CD39 (Fig. 3G–I) and CD52 was similar between the two groups.

### Humoral factors drove strong immune response of MAIT cells

**Figure 4:**
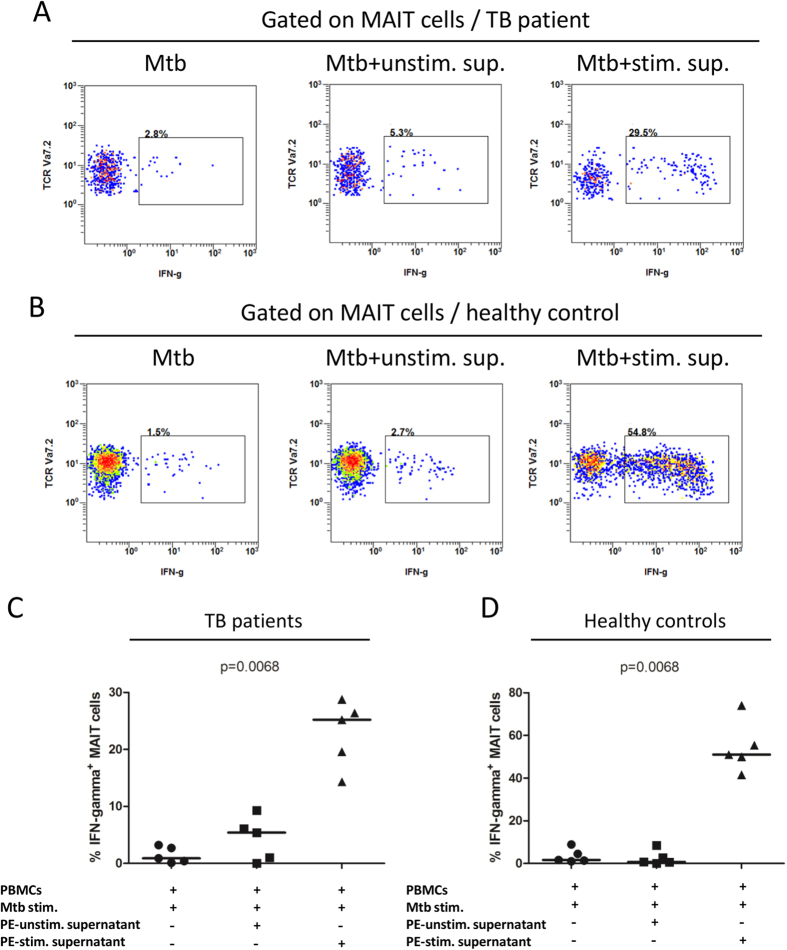
Influence of humoral factors on IFN-γ response of MAIT cells from PBMCs. (**A**) Representative flow plots showing IFN-γ production in MAIT cells from PBMCs of patient with TB under *Mtb* stimulation only (Mtb) (left), or with addition of centrifuged supernatant (100 μl) of unstimulated mononuclear cells (Mtb + unstim. sup.) (middle), or supernatants of *Mtb*-stimulated mononuclear cells from tuberculous pleural effusion (Mtb + stim. sup.) (right). (**B**) Representative flow plots showing IFN-γ production in MAIT cells from PBMCs of healthy control under same stimulation conditions as stated above. (**C**) IFN-γ response of MAIT cells in PBMCs from patients with TB (n = 5) under condition of *Mtb* stimulation alone (Mtb stim.), or with addition of centrifuged supernatants (100 μl) of unstimulated (PE-unstim. supernatant) or *Mtb* antigen-stimulated cells (PE-stim. supernatant) from tuberculous pleural effusions. (**D**) Supernatants of *Mtb*-stimulated cells from tuberculous pleural effusions drove strong IFN-γ response of MAIT cells in PBMCs from healthy controls (n = 5). Horizontal bars in the scatter plots indicate medians. Kruskal-Wallis test was used for statistical analysis among groups.

To understand whether soluble factors play a role in the immune response of MAIT cells, PBMCs from patients with TB were stimulated with *Mtb* lysates only, or together with centrifuged culture supernatants of unstimulated or *Mtb* antigen-stimulated mononuclear cells from tuberculous pleural effusions (Fig. 4A,C). Supernatants of *Mtb* antigen-stimulated cells from tuberculous pleural effusions drove strong IFN-γ response in MAIT cells from PBMCs of patients with TB (p = 0.0068), while supernatants of unstimulated mononuclear cells did not show such effect (Fig. 4A,C). Similar enhancing effect was also observed in MAIT cells from PBMCs of healthy controls (p = 0.0068) (Fig. 4B,D).

### Elevated immune response of MAIT cells from tuberculous pleural effusions depends on IL-2, IL-12 and IL-18

**Figure 5:**
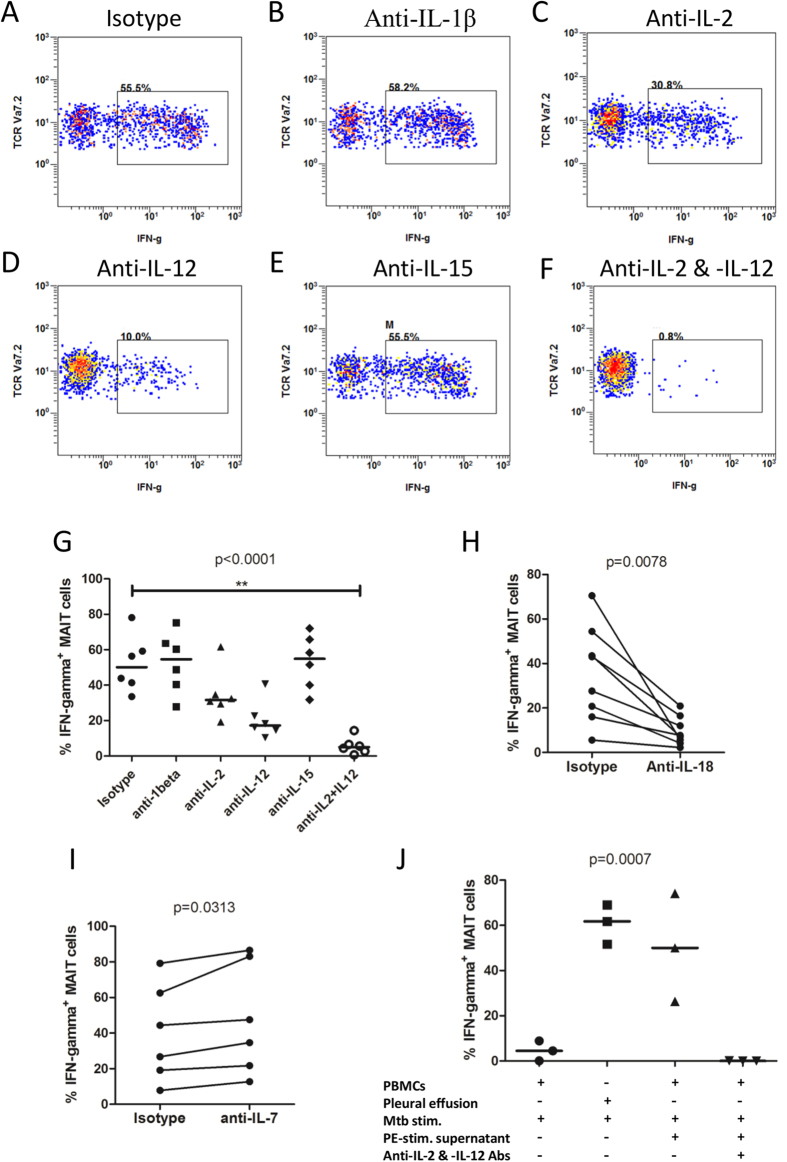
The role of cytokines on IFN-γ response of MAIT cells from tuberculous pleural effusions. (**A**–**F**) Representative flow plots showing IFN-γ production in *Mtb*-stimulated MAIT cells in the presence of isotype antibody control (**A**), or blocking antibody to IL-1β (**B**), IL-2 (**C**), IL-12 (**D**), IL-15 (**E**), or combined blocking antibodies of both IL-2 and IL-12 (**F**). (**G**) Reduced IFN-γ response in MAIT cells from tuberculous pleural effusions was observed when IL-2 and IL-12 were blocked with antibodies (n = 6). (**H**) Addition of IL-18 blocking antibody led to decreased IFN-γ production in MAIT cells (n = 8). (**I**) Blockade of IL-7 resulted in slightly higher IFN-γ response in MAIT cells (n = 6). (**J**) IFN-γ response in MAIT cells of PBMCs (PBMCs) in the presence of *Mtb* stimulation and centrifuged supernatants (100 μl) of *Mtb* antigen-stimulated cells from tuberculous pleural effusions was abolished by blocking antibodies to IL-2 and IL-12 (n = 3). Pleural effusion: mononuclear cells from tuberculous pleural effusions; Mtb stim.: *Mtb* antigen stimulation; PE-stim. supernatant: supernatants of *Mtb* antigen-stimulated cells from tuberculous pleural effusions; Anti-IL-2 & IL-12 Abs: combined blocking antibodies to IL-2 and IL-12. The Friedman’s test (p < 0.0001), followed by Dunn’s multiple comparison test was used in Fig. 5G. The non-parametric Wilcoxon signed-rank test was used for statistical analysis between groups in Fig. 5H,I. Kruskal-Wallis test was used for statistical analysis in Fig. 5J. Horizontal bars in the scatter plots indicate medians. **p < 0.01.

To determine whether cytokines drive the immune response of MAIT cells in tuberculous pleural effusions, blocking antibodies to IL-1β, IL-2, IL-12 and IL-15 were added during antigen stimulation of MAIT cells. Reduced IFN-γ response in MAIT cells from tuberculous pleural effusions was observed when IL-2 and IL-12 were blocked with specific antibodies (p < 0.0001), while blockade of IL-1β and IL-15 did not have significant effect (Fig. 5A–G). Addition of IL-18 blocking antibody led to decreased IFN-γ production in MAIT cells as well (p = 0.0078) (Fig. 5H). However, blockade of IL-7 resulted in slightly higher IFN-γ response in MAIT cells (p = 0.0313) (Fig. 5I).

To know whether IL-2 and IL-12 are responsible for enhancing effect of supernatants from *Mtb* antigen-stimulated cells, blocking antibodies to IL-2 and IL-12 were added to the culture medium during *Mtb* antigen stimulation of PBMCs, in the presence of stimulated supernatant. Blockade of both IL-2 and IL-12 nearly completely eliminated the enhancing effect driven by supernatant of *Mtb* antigen-stimulated cells from tuberculous pleural effusions (p = 0.0007) (Fig. 5J).

**Figure 6:**
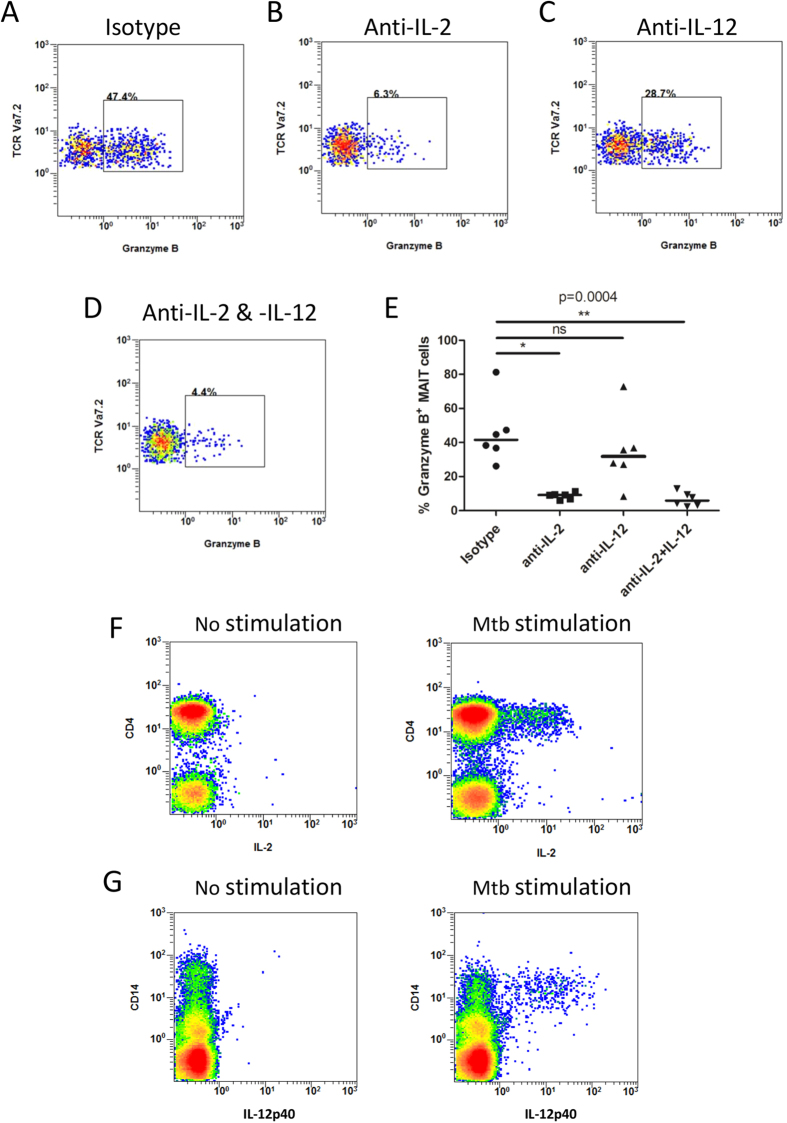
The role of cytokines on granzyme B production in MAIT cells from tuberculous pleural effusions. (**A**–**D**) Representative flow plots showing granzyme B production in *Mtb*-stimulated MAIT cells in the presence of isotype antibody control (**A**), or blocking antibody to IL-2 (**B**) and IL-12 (**C**), or combined blocking antibodies to both IL-2 and IL-12 (**D**). (**E**) Blockade of IL-2, but not IL-12, led to significantly reduced expression of granzyme B (n = 6). (**F**) Representative flow plots showing IL-2-producing cells in the tuberculous pleural effusions. Cells from tuberculous pleural effusions (n = 6) were not stimulated (left) or stimulated with *Mtb* lysates (right). All cells were gated and CD3^+^CD4^+^ T cells were identified as CD4^+^ T cells. (**G**) Representative flow plots showing IL-12p40-producing cells. Cells from tuberculous pleural effusions (n = 6) were not stimulated (left) or stimulated with *Mtb* lysates (right). All cells were gated and CD14^+^ cells were identified. The Friedman’s test (p < 0.0001), followed by Dunn’s multiple comparison test was used for statistical analysis among groups in Fig. 6E. Horizontal bars in the scatter plots indicate medians. *p < 0.05; **p < 0.01; ns: no significant difference.

Next, we examined whether granzyme B production in MAIT cells was affected by IL-2 and IL-12. Blockade of IL-2 led to significantly reduced expression of granzyme B in MAIT cells from tuberculous pleural effusions (p < 0.05), while blocking antibody to IL-12 alone did not show significant effect (Fig. 6).

To determine the source of IL-2 and IL-12, we investigated the phenotypes of IL-2- and IL-12p40-producing cells stimulated with *Mtb* antigens. Majority of IL-2-producing cells (94.50% ± 2.393, n = 6) in the tuberculous pleural effusions had phenotype of CD3^+^CD4^+^ (Fig. 6F), and most IL-12p40-producing cells (91.39% ± 3.024, n = 6) were CD14^+^ cells (Fig. 6G).

The concentration of IL-2, IL-12, IL-18 and IL-23 in supernatants from *Mtb*-stimulated blood PBMCs and cells from pleural effusions of TB patients were measured by Luminex method. The IL-2, IL-12, and IL-18 production was higher in supernatants of cells from pleural effusions than blood PBMCs, while IL-23 production was lower in supernatants of cells from pleural effusions; however, only the difference in IL-2 production was statistically significant (Supplemental Fig. 1).

Taken together, the results showed that enhanced production of cytokine and cytotoxic effector in MAIT cells from tuberculous pleural effusions was dependent on IL-2 produced predominantly by CD4^+^ T cells and/or IL-12p40 produced mainly by CD14^+^ cells.

### Elevated immune response of MAIT cells from tuberculous pleural effusions correlated with γc expression

**Figure 7:**
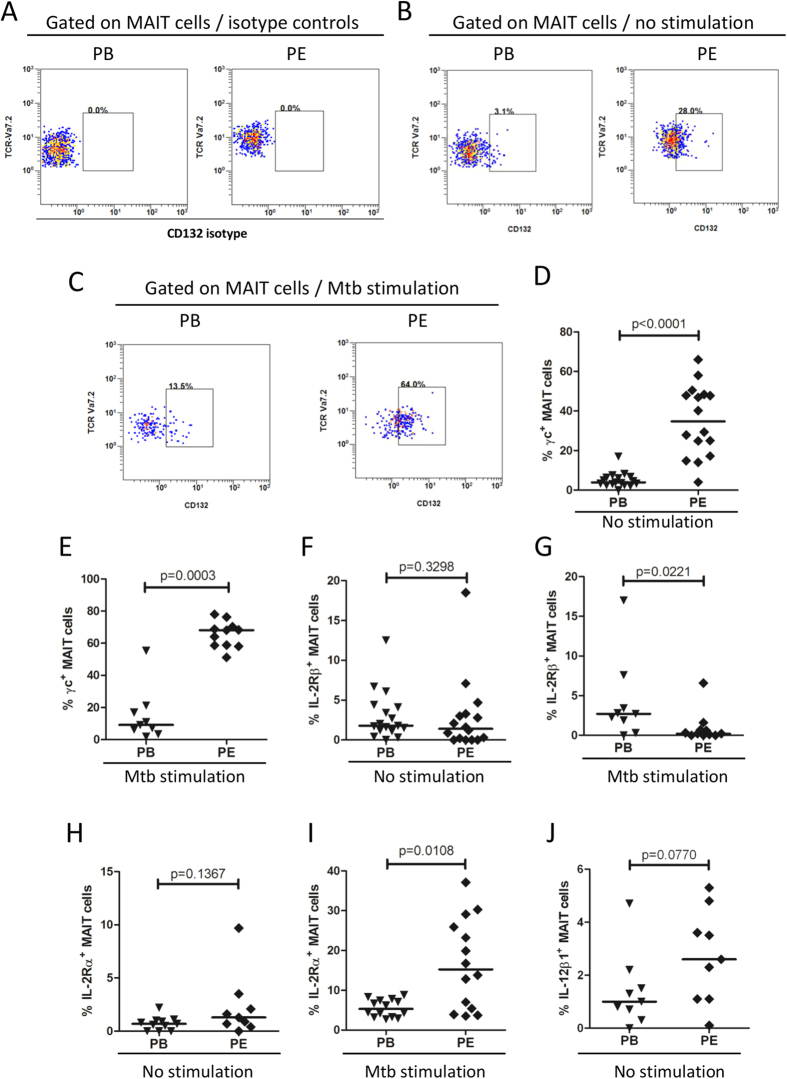
IL-2 and IL-12 receptor expression on MAIT cells from peripheral blood and tuberculous pleural effusions. (**A**) Representative flow plots showing γc (CD132) isotype control antibody staining of MAIT cells from peripheral blood (PB) and tuberculous pleural effusion (PE). (**B**) Representative flow plots showing expression of γc receptor (CD132) on MAIT cells in the absent of antigen stimulation. (**C**) Representative flow plots showing expression of γc receptor (CD132) on *Mtb*-stimulated MAIT cells from peripheral blood (PB) and tuberculous pleural effusion (PE). (**D**–**E**) MAIT cells from tuberculous pleural effusions exhibited significantly higher expression of γc receptor (CD132) than those from peripheral blood both in the absent of antigen stimulation (**D**) and after *Mtb* stimulation (**E**). (**F**–**G**) The expression of IL-2Rβ on MAIT cells in the absent of antigen stimulation (**F**) or after *Mtb* stimulation (**G**). (**H**–**I**) The expression of IL-2Rα on MAIT cells in the absent of antigen stimulation (**H**) or after *Mtb* stimulation (**I**). (**J**) The expression of IL-12Rβ1 on MAIT cells in the absent of antigen stimulation. Horizontal bars in the scatter plots indicate medians. The nonparametric Mann-Whitney test was used for statistical analysis between groups.

It is known that IL-2 functions through three receptors, IL-2Rα, IL-2Rβ and γc. To understand whether IL-2 signals through these receptors on MAIT cells, the expression of the three receptors on MAIT cells from tuberculous pleural effusions and peripheral blood was examined. MAIT cells from tuberculous pleural effusions exhibited significantly higher expression of γc receptor than those from peripheral blood both in the absence of *Mtb* antigen stimulation (p < 0.0001) and after *Mtb* stimulation (p = 0.0003) (Fig. 7A–E). The expression of IL-2Rβ and IL-2Rα on MAIT cells was similar between the two groups in the absence of *Mtb* antigen stimulation (Fig. 7F,H). After *Mtb* antigen stimulation, MAIT cells from tuberculous pleural effusions had lower expression of IL-2Rβ (p = 0.0221) and higher expression of IL-2Rα (p = 0.0108) than those from peripheral blood (Fig. 7G–I).

IL-12 receptor is a heterodimer formed by IL-12Rβ1 and IL-12Rβ2. Both IL-12Rβ1 and IL-12Rβ2 were expressed on MAIT cells, and IL-12Rβ1 expression was higher in MAIT cells from tuberculous pleural effusions than from peripheral blood in the absence of *Mtb* stimulation, but the difference was not statistically significant (Fig. 7J). IL-12Rβ2 expression was similar between MAIT cells from tuberculous pleural effusions and from peripheral blood (data not shown).

To know the role of IL-18 receptor α (IL-18Rα) on MAIT cells, the expression of IL-18Rα was investigated. IL-18Rα was expressed on most MAIT cells from both tuberculous pleural effusions (91.25% ± 4.994) and from peripheral blood (83.98% ± 5.589) in the absence of antigen stimulation, and there is no statistical difference in frequency of IL-18Rα-expressing MAIT cells between the two groups (data not shown).

### Blockade of γc and IL-2Rβ receptors resulted in reduced immune response of MAIT cells

**Figure 8:**
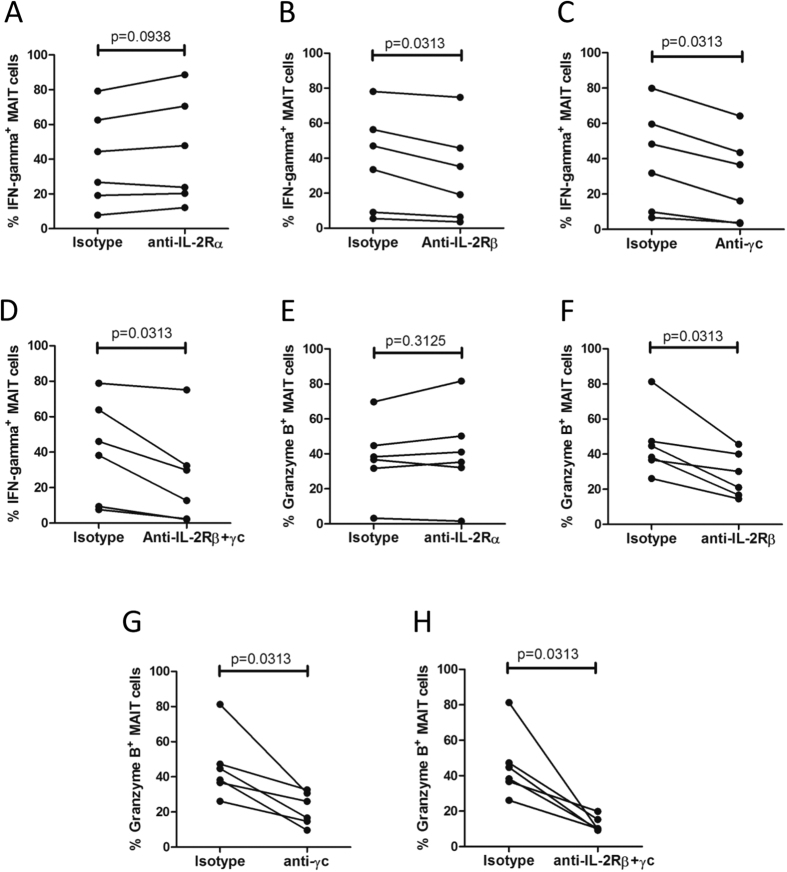
Blockade of γc and IL-2Rβ receptors resulted in reduced immune response of MAIT cells from tuberculous pleural effusions. (**A**) Blockade of IL-2Rα did not have significant effect on IFN-γ response in MAIT cells stimulated with *Mtb* lysates. (**B**–**C**) Reduced IFN-γ response in MAIT cells was observed when IL-2Rβ (**B**) and γc receptors (**C**) were blocked with antibodies respectively. (**D**) Blockade of both IL-2Rβ (**B**) and γc receptors resulted in decreased IFN-γ response in MAIT cells. (**E**) Blockade of IL-2Rα did not have significant effect on granzyme B production in MAIT cells. (**F**–**G**) Blockade of IL-2Rβ (**F**) or γc receptor (**G**) led to significantly reduced production of granzyme B in MAIT cells. (**H**) Combined blocking of both IL-2Rβ and γc receptors resulted in even more reduced production of granzyme B. The non-parametric Wilcoxon signed-rank test was used for statistical analysis.

To determine the role of IL-2 receptors on immune response of MAIT cells, blocking antibodies to IL-2Rα, IL-2Rβ and γc were used. Blockade of IL-2Rα did not have significant effect on IFN-γ response in MAIT cells from tuberculous pleural effusions (p = 0.0938) (Fig. 8A). However, reduced IFN-γ production in MAIT cells was observed when IL-2Rβ (p = 0.0313) and/or γc receptors (p = 0.0313) were blocked with specific antibodies (Fig. 8B–D).

The role of IL-2 receptors on granzyme B production was also investigated. Similar to IFN-γ response, blockade of IL-2Rβ (p = 0.0313) and γc receptors (p = 0.0313) led to significantly reduced production of granzyme B in MAIT cells from tuberculous pleural effusions, while blocking antibody to IL-2Rα did not show significant effect (p = 0.3125) (Fig. 8E–G). Combined blockade of both IL-2Rβ and γc receptors resulted in even more reduced production of granzyme B (p = 0.0313) (Fig. 8H).

### Signaling pathways involved in FN-γ response of MAIT cells from tuberculous pleural effusions

**Figure 9:**
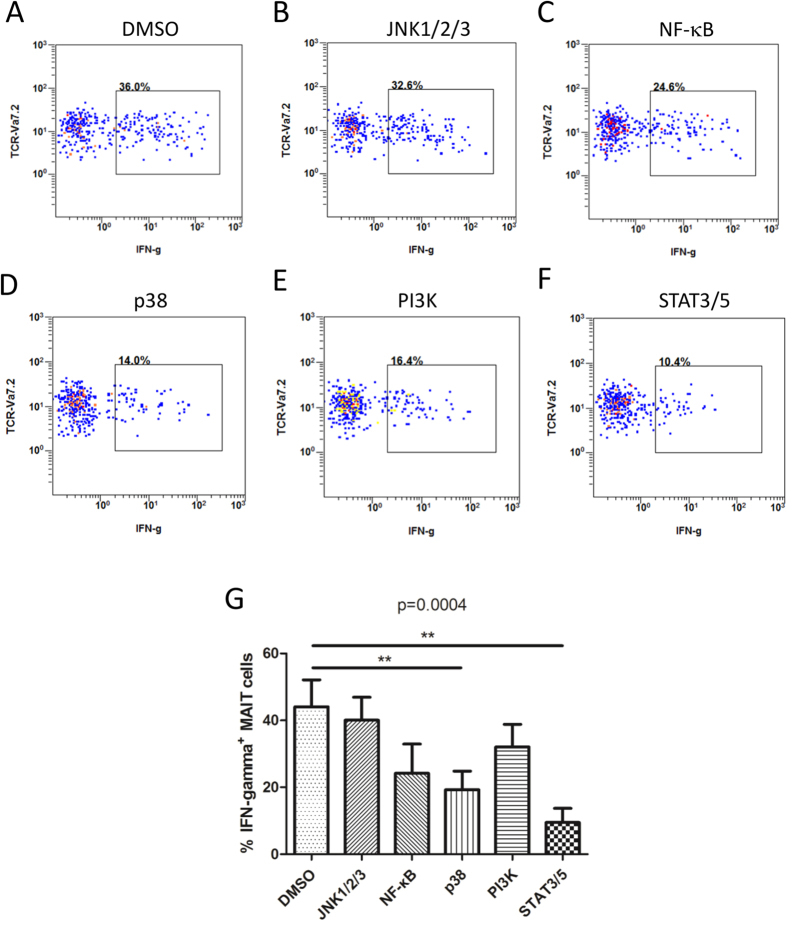
Blockade of signaling pathways with small molecule inhibitors. (**A**–**F**) Representative flow plots showing IFN-γ production in *Mtb*-stimulated MAIT cells in the presence of solvent DMSO (**A**), or small molecule inhibitors to JNK1/2/3 (**B**), NF-κB (**C**), p38 (**D**), PI3K (**E**), or STAT3/STAT5 (**F**). (**G**) Inhibition effect of small molecule inhibitors on IFN-γ production in *Mtb*-stimulated MAIT cells from tuberculous pleural effusions (n = 8). The Friedman’s test (p = 0.0004), followed by Dunn’s multiple comparison test was used for statistical analysis among groups. **p < 0.01.

To know the signaling pathways involved in immune response of MAIT cells from tuberculous pleural effusions, small molecule inhibitors were added to culture medium of cells during *Mtb* antigen stimulation. Inhibitors of JNK1/2/3 (SP600125), NF-κB (BAY11-7802) and PI3 Kinase (p100α/δ/β) (LY294002) did not show significant effect on IFN-γ production in MAIT cells compared with DMSO solvent control (Fig. 9). However, reduced production of IFN-γ was observed when inhibitors of p38 MAPK (SB203580) and STAT3/5 (SH-4-54) were added to culture medium of MAIT cells respectively (p < 0.01) (Fig. 9). The results suggested that STAT3/5 and p38 signaling pathways might involve in IFN-γ response in MAIT cells from tuberculous pleural effusions.

## Discussion

Tuberculous pleurisy is believed to be caused by direct infection of *Mtb* and could be used as a model to study immune response of immune cells at the site of TB infection^2^. In this study, we compared the phenotypes and immune response of MAIT cells from tuberculous pleural effusions and peripheral blood, and found that MAIT cells in tuberculous pleural effusions, the site of *Mtb* infection, had greatly elevated immune response to *Mtb* antigens compared with those from peripheral blood. The elevated immune response correlated with increased γc receptor expression on MAIT cells.

MAIT cells are depleted from peripheral blood in a variety of diseases, including TB^5^,^6^,^24^,^25^, AIDS^26^,^27^,^28^, type 2 diabetes^29^, and systemic lupus erythematosus^30^. During bacterial infection, MAIT cells might be recruited to the sites of infection and provide protection^6^,^20^. It is interesting to understand the phenotypes and functional differences between MAIT cells from local sites of infection and from peripheral blood.

Protective immunity against TB is associated with cytokines and cytotoxic effectors provided by T cells^31^,^32^,^33^,^34^,^35^,^36^. MAIT cells not only secret cytokines, but also have cytotoxic effect on infected cells, which probably responsible for its anti-microbial function^5^,^6^,^7^,^8^. Our study found that MAIT cells from tuberculous pleural effusions exhibit highly elevated production of IFN-γ and IL-17F cytokines and also cytotoxic effector granzyme B, compared with those from peripheral blood. This is in contrast with the observation that MAIT cells in peripheral blood from patients with active TB showed defects in activation and cytokine production^24^,^25^. Therefore, unlike in peripheral blood, MAIT cell functions are greatly enhanced at the site of TB infection, most likely by signals provided by other cells through humoral factors, suggesting that microenvironment where MAIT cells are located is critical for their function.

The level of IFN-γ response in MAIT cells from tuberculous pleural effusions was correlated negatively with the extent of TB infection, and this finding suggests that patients with serious TB might have depressed IFN-γ production in MAIT cells at the site of infection.

To understand whether cytokines drive the enhanced immune response of MAIT cells from tuberculous pleural effusions, we investigated a number of cytokines that are known to be produced by immune cells upon *Mtb* infection^37^,^38^. Blockade of cytokines IL-2, IL-12 and IL-18 resulted in significantly reduced production of IFN-γ and/or granzyme B in MAIT cells. This is consistent with previous reports that addition of IL-2 or IL-12 could enhance IFN-γ response in MAIT cells to *Mtb* antigens^39^, and MAIT cells are specifically activated by IL-12 and/or IL-18^40^,^41^,^42^. It is not clear why blockade of IL-7 resulted in slightly increased IFN-γ production, since IL-7 was shown to active MAIT cells^43^. To our knowledge, the role of IL-2 on MAIT cell function at the site of *Mtb* infection has not been reported so far. These results suggest that the acquired T cell immunity could not only produce effector molecules against *Mtb* infection by itself, but also could enhance MAIT cell function via IL-2 production to have combined effect in controlling TB infection.

IL-2 is a pleiotropic cytokine that is primarily produced by active CD4^+^ T cells, and it is also produced, although at lower levels, by activated dendritic cells (DCs), CD8^+^ T cells, NKT cells, and mast cells^44^. It signals through intermediate affinity receptor of IL-2Rβ/γc chains expressed mainly on memory T cells and NK cells, or high affinity receptor of IL-2Rα/IL-2Rβ/γc complex^45^. IL-2Rα alone does not contribute to signaling due a short cytoplasmic tail, and IL-2Rβ/γc chains are responsible for signaling^46^. Our study showed that in tuberculous pleural effusions, IL-2 was predominantly produced by CD4^+^ T cells in response to *Mtb* antigen stimulation. Although MAIT cells have expression of all three IL-2 receptor chains, γc chain was the only one that had high levels of expression on MAIT cells from tuberculous pleural effusions both in the absence and presence of *Mtb* stimulation, compared with those from peripheral blood. It is not clear why expression of γc receptor and IL-2Rβ in MAIT cells from tuberculous pleural effusions after stimulation was different.

IL-12 is an inflammatory cytokine produced mainly by dendritic cells (DCs), monocytes and macrophages, and it can induce IFN-γ production by T cells and NK cells and promote Th1 differentiation^47^. In MAIT cells, inhibition of intracellular growth of *M. bovis* BCG in macrophages is dependent upon IFN-γ, as well as IL-12 signal^23^. It has been reported that IL-12 can rescue the exhausted antigen-specific CD8^+^ T cells in chronic hepatitis B infection^48^. IL-12 receptor consists of IL-12β1 and IL-12β2^47^. Individuals with IL-12β1 deficiency are more likely to have severe infection with mycobacteria and salmonella^49^. Our study found that IL-12β1 is expressed on MAIT cells from tuberculous pleural effusions, however, the role of IL-12β1 on MAIT cell function remains to be elucidated. The expression of IL-18Rα on MAIT cells from tuberculous pleural effusions and peripheral blood was similar, which suggests that IL-18Rα was probably not associated with enhanced MAIT cell immune responses in tuberculous pleural effusions.

Jak/STAT signaling pathway has wide range of functions, and contains 4 Jaks (JAK1, 2, 3, and TYK2) and 7 STATs (STAT1, 2, 3, 4, 5a, 5b, and 6)^50^. STATs can be phosphorylated by Jaks and result in dimerization, translocation to nuclear and binding to specific motifs of DNA, leading to enhanced transcription of target genes. It has been reported that engagement of IL-2 receptor by IL-2 leads to activation of predominately STAT5 pathway^44^. Interaction of IL-12 with IL-12 receptor results in activation of STAT4 that enhances transcription of IFN-γ and other target genes^47^. p38, together with JNKs and ERKs, belongs to mitogen-activated protein (MAP) kinases, and includes p38α, p38β, p38γ, and p38δ^51^. It has multiple functions in difference cell types, and has been reported to involve in differentiation of Th1 cells and production of IFN-γ^52^. Our result suggests that STAT3/STAT5 and p38 pathways might be critical in regulating IFN-γ response of MAIT cells from tuberculous pleural effusions.

In summary, MAIT cells in tuberculous pleural effusions had greatly enhanced IFN-γ, IL-17F and granzyme B response compared with those in peripheral blood. The level of IFN-γ response in MAIT cells from tuberculous pleural effusions was inversely correlated with the extent of TB infection. The elevated immune response of MAIT cells from tuberculous pleural effusions was mainly dependent on IL-2 and IL-12 produced predominantly by CD4^+^ T cells and CD14^+^ cells respectively, and correlates with γc receptor expression.

## Materials and Methods

### Human subjects

Forty-two patients with tuberculous pleurisy were recruited and their clinical characteristics were summarized in Table 1. They were diagnosed according to the 1990 edition of the Diagnostic Standards and Classification of Tuberculosis, published by the American Lung Association. Among the 42 patients with tuberculous pleurisy, 11 patients had only tuberculous pleurisy without lesions found in lung and other organs, 12 were accompanied with pulmonary TB with lesions located within one lung, 8 had pulmonary TB that affected both lungs, and 11 had both pulmonary TB and other extrapulmonary TB. All patients were HIV-negative.

Nine healthy controls (male/female: 6/3, mean age: 37.33 ± 1.922) were randomly recruited from individuals undergoing annual health check-ups at the clinics of the 309th Hospital, with following inclusion criteria: (1) no fever, cough or other signs of active TB; (2) with normal physical examination result and normal radiography; (3) without HIV infection.

The study was approved by the Ethics Committee of the 309^th^ Hospital. The institutional review board for human studies approved the protocols. Informed consent was obtained from all subjects. All experiments were performed in accordance with relevant guidelines and regulations.

### Antibodies

Monoclonal antibodies to human TCR Vα7.2-FITC or -PE (clone 3C10), CD161-APC or –PE/Cy7 (clone HP-3G10), CD4-FITC (clone OKT4), CD8α-PE/Cy5 (clone HIT8a), CD25 (IL-2Rα)-FITC (clone BC96), CD26-FITC (clone BA5b), CD27-FITC (clone O323), CD39-FITC (clone A1), CD45RO-PE/Cy5 (clone UCHL1), CD62L-FITC (clone DREG-56), CD122-PE (IL-2Rβ) (clone TU27), CD132 (common γ chain)-APC (clone TUGh4), IL-17F-Alexa Fluor 488 (clone poly5166), CD218a (IL-18Rα)-FITC (clone H44), anti-IL-12p40-PE (clone C11.5), and LEAF purified anti-human CD28 monoclonal antibody (clone CD28.2) were purchased from BioLegend (San Diego, CA, USA). Antibodies of anti-human CD3-FITC, -PE-CF594 or -PE-Cy7 (clone UCHT1), anti-granzyme B-FITC (clone GB11), anti-TNF-α-FITC or -APC (clone MAb11), anti-IL-12Rβ1-APC (clone 2.4E6), anti-IL-12Rβ2-PE (clone 2B6/12beta2), and the cytofix/cytoperm fixation/permeabilization kit were obtained from BD Biosciences (San Diego, California, USA). Anti-human IFN-γ-FITC (clone #25723), anti-IL-2-PE (clone MQ1-17H12), anti-human IL-1β (clone #2805), anti-IL-2 (clone #5334), anti-IL-7 (clone #7417), anti-IL-12p70 (clone #24910), anti-IL-15 (clone #34505), anti-IL-18 (clone #125-2H), anti-IL-2Rα (clone #22722), anti-IL-2Rβ (clone #27302) and anti-common γ chain (IL-2Rγ) receptor antibodies (clone #633162) were obtained from R&D Systems (Minneapolis, MN, USA). Isotype-matched control antibodies were purchased from the corresponding suppliers mentioned above to determine the background level of staining, or to be used as controls for blocking experiment.

### Flow cytometry

Mononuclear cells were purified from tuberculous pleural effusions and peripheral blood mononuclear cells (PBMCs) were purified from peripheral blood by density gradient centrifugation using Ficoll-Paque PLUS (GE Healthcare Bio-Sciences, Pittsburgh, PE, USA) as described previously^24^,^53^. For surface marker staining, fluorochome-labeled monoclonal antibodies were mixed with mononuclear cells or PBMCs, and incubated at 4 °C for 30 min in the dark. Cells were analyzed with Beckman CXP software on a FC-500 flow cytometer (Beckman Coulter, Brea, CA, USA). Appropriate isotype-matched control antibodies were used to determine background levels of staining. Live/dead cells of lymphocytes were determined by staining with 7-AAD viability staining solution (BioLegend), and the ratio of dead cells was normally below 1.2% of total lymphocytes.

### Antigen stimulation

A total of 1 × 10^6^ mononuclear cells from tuberculous pleural effusions or PBMCs were cultured in AIM V^®^ serum-free medium (Gibco, Life Technologies, Grand Island, NY, USA). The cells were stimulated with *M. tuberculosis* strain H37Rv sonicated lysates at a concentration of 10 μg/ml overnight at 37 °C, in the presence of 1 μg/ml anti-human CD28 antibody (BioLegend, San Diego, CA, USA). For detection of intracellular cytokines and granzyme B, brefeldin A (BD Biosciences) was added to the cell suspension 4 hours before staining of the cells, as described previously^24^. The cytokine concentration in the supernatants of cells from pleural effusions and blood PBMCs was measured by Luminex method with Human Cytokine Panel kit (eBioscience, San Diego, CA, USA).

### Intracellular cytokine and granzyme B staining

For intracellular cytokine staining, stimulated cells were stained first with fluorochome-labeled monoclonal antibodies to surface markers. After being permeabilized with cytofix/cytoperm fixation/permeabilization buffer (BD Biosciences), the cells were incubated with fluorochrome-labeled monoclonal antibody to human IFN-γ, IL-17F, TNF-α, or granzyme B for 30 minutes at 4 °C. Appropriate isotype-matched control antibodies were used to determine background levels of staining. The expression of cytokines and granzyme B was analyzed by an FC-500 flow cytometer (Beckman Coulter).

### Blockade of cytokines and receptors

Blocking monoclonal antibodies to human IL-1β, IL-2, IL-7, IL-12p70, IL-15, IL-18, IL-2Rα, IL-2Rβ, or γc receptor (R&D Systems, Minneapolis, MN, USA), at a concentration of 10 μg/ml, were added to the culture medium of mononuclear cells from tuberculous pleural effusions or PBMCs one hour before addition of *Mtb* H37Rv lysates respectively, and the cells were incubated at 37 °C incubator with 5% CO_2_ overnight. Mouse IgG1 κ and IgG2b κ isotype control antibodies were used as controls.

### Blockade with small molecule inhibitors

Small molecule inhibitors, including SP600125 (JNK1/2/3 inhibitor), BAY11-7802 (NF-κB inhibitor), SB203580 (p38 MAPK inhibitor), LY294002 (PI3 Kinase p100α/δ/β inhibitor) and SH-4-54 (STAT3/5 inhibitor) (all from Selleck Chemicals, Houston, Texas, USA), were added to the culture medium of mononuclear cells from tuberculous pleural effusions one hour before addition of *Mtb* lysates, and cells were incubated at 37 °C incubator with 5% CO_2_ overnight. The solvent DMSO at appropriate concentration was used as controls. The final concentration used was 2 μM for SP600125, 1 μM for BAY11-7802, 10 μM for SB203580, 10 μM for LY294002, and 5 μM for SH-4-54.

### Statistical analysis

The Mann-Whitney test, Kruskal-Wallis test, Friedman’s test and Dunn’s multiple comparison test, Wilcoxon signed-rank test and correlation analysis were used for statistical analysis with Prism program version 5.01 (GraphPad Software, San Diego, CA, USA), as indicated in the text. All tests were two-tailed, and *p* < 0.05 was considered significant.

## Additional Information

**How to cite this article**: Jiang, J. *et al.* Enhanced immune response of MAIT cells in tuberculous pleural effusions depends on cytokine signaling. *Sci. Rep.* **6**, 32320; doi: 10.1038/srep32320 (2016).

## Supplementary Material

Supplementary Information

## Figures and Tables

**Table 1:** Demographic and clinical characteristics of patients with active TB.

	Patients with active TB
N (male/female)	42 (33/9)
Age (mean ± SD)	33.93 ± 2.666
Tuberculous pleurisy	42
With pTB^1^: lesions in one lung	12
With pTB^1^: lesions in both lungs	8
With pTB^1^ and extrapulmonary TB in other organs	11
Adenosine deaminase: >40 u/L	35
Adenosine deaminase: 35–40 u/L	7

^1^pTB: pulmonary tuberculosis.
