# Supplementary material for: Enhanced immune response of MAIT cells in tuberculous pleural effusions depends on cytokine signaling
Source: Sci Rep. 2016 Sep 2;6:32320. doi: 10.1038/srep32320 (PMC5009363; doi:10.1038/srep32320)
Supplement: Supplementary Information [file srep32320-s1.doc]

**Supplementary file**

**Enhanced immune response of MAIT cells in tuberculous pleural effusions depends on cytokine signaling**

Jing Jiang^1,3,4^, Xinchun Chen^1,2^*, Hongjuan An^3^, Bingfen Yang^3^, Fuping Zhang^4^, Xiaoxing Cheng^3^*

^1^Shenzhen Key Laboratory of Infection and Immunity, Shenzhen Third People's Hospital, Guangdong Medical College, Shenzhen, Guangdong, China

^2^Department of Pathogen Biology, School of Medicine, Shenzhen University, Shenzhen, Guangdong, China

^3^Division of Research, Institute of Tuberculosis, 309^th^ Hospital, Beijing, China

^4^Institute of Microbiology, Chinese Academy of Sciences, Beijing, China

*Corresponding authors (Xinchun Chen and Xiaoxing Cheng)

Supplemental Figure 1.


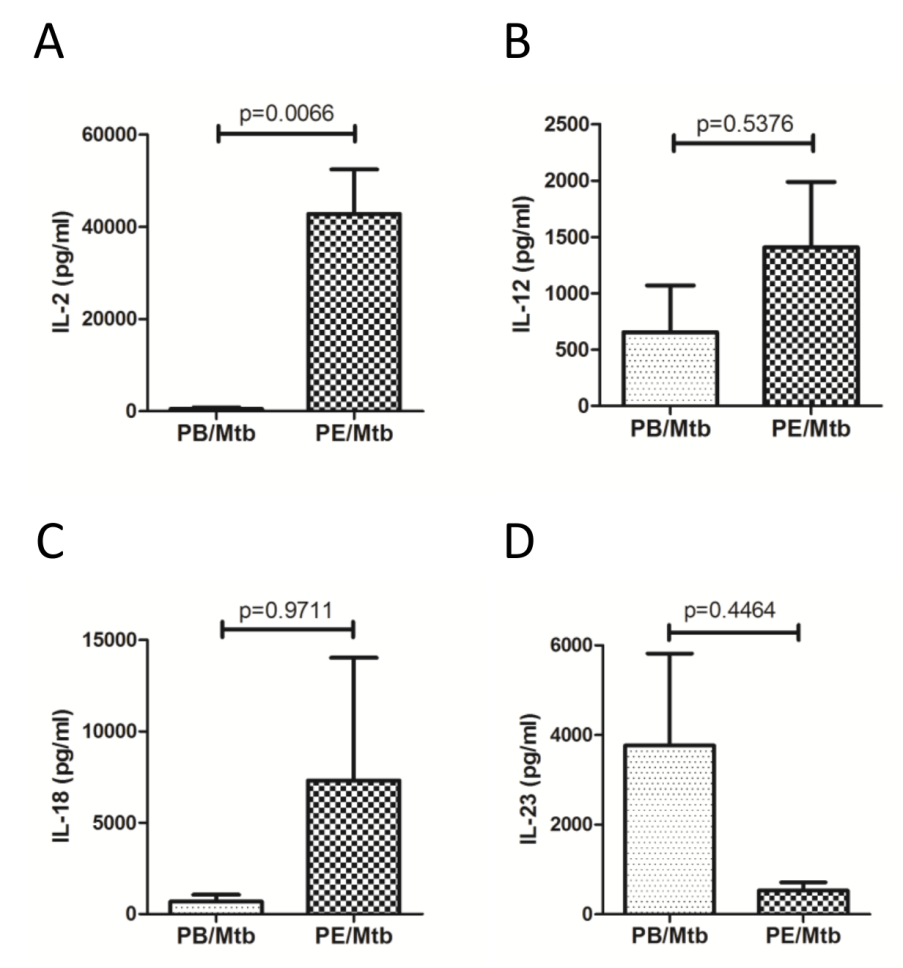


**Figure legend**

Supplemental Figure 1. IL-2, IL-12, IL-18 and IL-23 production in supernatants from *Mtb*-stimulated blood PBMCs and cells from tuberculous pleural effusions. The concentration of IL-2 (A), IL-12 (B), IL-18 (C) and IL-23 (D) in supernatants of *Mtb*-stimulated blood PBMCs (PB/Mtb, n=8) and cells from tuberculous pleural effusions (PE/Mtb, n=13) was measured by Luminex method. The nonparametric Mann-Whitney test was used for statistical analysis between groups.
